# Use of the Fractal Dimension to Differentiate Epithelium and Connective Tissue in Oral Leukoplakias

**DOI:** 10.3390/cancers14112697

**Published:** 2022-05-30

**Authors:** Yolanda Guerrero-Sánchez, Francisco Gómez García, Cintia M. Chamorro-Petronacci, José M. Suárez-Peñaranda, Mario Pérez-Sayáns

**Affiliations:** 1 Department of Human Anatomy and Psicobiology, University of Murcia, 30100 Murcia, Spain; yolanda.guerreros@um.es; 2 Research Virgen de la Arrixaca Clinical University Hospital, IMIB-Arrixaca, University of Murcia, 30100 Murcia, Spain; fjgomez@um.es; 3 Health Research Institute Foundation of Santiago (FIDIS), (ORALRES Group) Oral Medicine, Oral Surgery and Implantology Unit, Faculty of Medicine and Dentistry, University of Santiago de Compostela (MedOralRes Group), 15782 Santiago de Compostela, Spain; perezsayans@gmail.com; 4 Pathological Anatomy Service, University Hospital Complex of Santiago (CHUS), 15782 Santiago de Compostela, Spain; jm.penaranda@gmail.com

**Keywords:** Oral leukoplakia, oral cancer, fractal dimension

## Abstract

**Simple Summary:**

Dysplasia grade identification is the only consolidated factor by which to evaluate the risk of developing oral cancer from oral leukoplakia lesions. An objective manner to determine dysplasia grade is still lacking and this has prompted our research. Our findings can help dentists and pathologists to predict oral leukoplakia prognosis with a non-invasive, easy-to-use tool, using the fractal dimension as invariant.

**Abstract:**

Background: Oral leukoplakia (OL) is considered one of the most common potentially malignant oral disorders (OPMD), with a verified increased risk of developing oral cancer. The identification of the dysplasia grade (low–high) is the only consolidated factor used to evaluate this risk. The objective of this study was to verify the role of the fractal dimension (FD) in assessing this dysplasia. Methods: To begin, 29 OL and 10 normal oral mucosa (NOM) biopsies were retrieved for FD analysis of the epithelial (***dime***) and the connective (***dimc***) tissue. Results: In the OL group, the median value of dime is higher (1.67, IQR = 0.12) than for the NOM group (1.56, IQR = 0.08), with statistically significant differences (Wilcoxon test, *p* = 0.0031). There were no differences in relation to dimc. Significant differences were observed between the non-dysplasia vs. high-grade (*p* = 0.0156) and low-grade vs. high-grade (*p* = 0.0049) groups. No significant differences were identified in relation to dimc for the different degrees of dysplasia. For a cut-off point of 1.44 of dime, a specificity of 96.6% was obtained, a sensitivity of 100%, and an AUC = 0.819 (*p* = 0.003). Conclusions: FD at the level of the epithelium may be used as a diagnostic tool in OL.

## 1. Introduction

Currently, oral leukoplakia (OL), defined as “a predominantly white plaque of questionable risk having excluded (other) known diseases or disorders that carry no increased risk for cancer”, is considered one of the most common oral potentially malignant disorders (OPMD). These OPMD include any abnormality of the oral mucosa that is associated with a statistically increased risk of developing oral cancer. OL carries an increased risk of cancer development near the area of the lesion or elsewhere in the oral cavity or the neck region [1,2,3,4].

Given the high incidence of head and neck cancers (3–4%), compared to cancer in general, it is vitally important to monitor premalignant lesions when searching for changes indicative of malignant transformation, in order to establish an early diagnosis [5]. The diagnosis is obtained through biopsy; the pathological analysis indicates the degree of dysplasia of the lesion, by contemplating the different alterations that occur in the tissue [6]. Kujan, et al., based on the 2005 WHO (i.e., World Health Organization) criteria for diagnosing dysplasia, developed a binary classification system of dysplasia (high and low grade) based on the combination of architectural and cytological changes [7,8]. It is then that we differentiate between high-grade dysplasias, which would have a greater capacity to become malignant, and low-grade dysplasias, which would be those with less potential for malignancy [9,10]. This binary classification system is currently used without differentiating the levels within the tissue.

The word fractal means “break” and since its introduction in the 1970s by the mathematician B. Mandelbrot, it has been used to designate a class of objects characterized by presenting patterns of self-similarity at different levels of scale. That is, by successively enlarging a fractal object, another example appears that is similar to the original object (see [11,12]). These types of structures appear in various contexts in nature and medicine (dentistry, ophthalmology, etc.), as well as in various lines of study in mathematics, physics, and economics, to name a few. In this sense, it should be noted that the study and analysis of fractal patterns have originated highly active lines of research in recent years due to the identification of new fractal structures [13,14]. The basic tool for studying the irregular nature of an object is the so-called *fractal dimension* (FD), which is a dimensionless topological invariant that provides very useful information on the complexity presented by the object of study when it is examined in different levels of detail. Although there are several definitions of FD, the box-counting model is often used in health science practice since its empirical estimation is feasible in practical applications [15]. Its origin dates back to the 1930s when it was proposed by Pontrjagin and Schnirelman [16]. This mathematical tool is used constantly at the radiographic level to study the bone trabeculate with the development of image treatment methods, such as that by White and Rudolph [17]. This method applies segmentation processes and filters to calculate the fractal dimension values, after eliminating artifacts and precisely defining the area to be studied.

The diagnosis of OL, as we said earlier, is established with a biopsy, obtaining a tissue sample that has a characteristic architecture. This architecture can be considered to be a fractal since it meets the requirement of being a mathematical invariant, maintaining the structure of epithelium, connective tissue, and cell layers [18,19,20,21].

The objective of this study was to verify if the FD can become an objective tool to assess both architectural and cytological changes in the oral mucosa. We also aim to establish whether, at the macroscopic level, FD is also a value that demonstrates sensitivity in terms of the changes produced at the level of limits in the surface and volume of the lesion, among others, which could be indicators of malignancy.

## 2. Materials and Method

### 2.1. Design of the Study

We carried out a multicenter study, between the University of Santiago de Compostela and the University of Murcia, of a longitudinal retrospective nature, using a sample of 39 patients with OL and 10 normal oral mucosa (NOM) biopsies from different areas of the oral cavity. The study has obtained a certificate from the bioethics committee of the University of Santiago de Compostela, with the registration code 2019/271, whence the samples were obtained. All procedures were carried out with the proper understanding and written consent of the subjects, in accordance with the Declaration of Helsinki and following the guidelines of the STROBE guide.

### 2.2. Computation of the Sample Size

Since there are few previous studies based on the fractality of OLs where significant results were obtained, we consider this study pioneering, laying the foundations for subsequent analyses that could permit power calculations and sample estimates.

### 2.3. Type of Samples and Processing

Samples were collected from January 2015 to June 2020 from patients treated in the Oral Medicine service unit at the Faculty of Dentistry (University of Santiago de Compostela). By sample, we mean: (1) a biopsy of the lesion area and (2) a digital photographic image of the biopsied area. The samples were stained with hematoxylin and eosin and the slides were digitized by the image service of the University of Murcia (SACE) for subsequent analysis.

### 2.4. Inclusion and Exclusion Criteria

The inclusion criteria for OL samples were: (1) a confirmed clinical and histopathological diagnosis of OL, according to the WHO 2017 criteria, (2) a follow-up period of at least two years, and (3) patients older than 18 years. The exclusion criteria were: (1) samples from patients who did not sign the informed consent or whose clinical data could not be extracted from the medical history, and (2) patients with white keratotic lesions, oral lichenoid lesions, oral lichen planus, and other lesions similar to OL.

Controls for NOM were selected from patients without OPMD who underwent the surgical extraction of third molars on the basis of the same criteria and were paired by the average age of OL samples (±7 years). In this way, in each control a fragment of NOM was obtained surrounding the target teeth, of approximately 5 × 5 mm in size; a non-inflamed appearance in this area was a prerequisite for inclusion.

### 2.5. Variables

The clinical variables were collected and defined, based on the above criteria, using the pathological anatomy report, then we allocated them to different groups according to the dysplasia variable: no dysplasia, low-grade dysplasia, or high-grade dysplasia. The clinical and pathological variables involved in the statistical analysis are described in Table 1 and Table 2 and are divided into quantitative and qualitative variables. **Quantitative variables**: (i) Fractal dimension of the epithelial tissue (***dime***). (ii) Fractal dimension of connective tissue (***dimc***). (iii) Age (**age**). (iv) Lesion size (**size**). (v) Number of lesions (**no.**). **Qualitative variables**: (i) Level of dysplasia (**dysplasia**), with 3 levels: 1. No dysplasia, 2. Low-grade dysplasia, 3. High-grade dysplasia. (ii) Location of biopsy (**location**), with 6 levels: 1. Tongue, 2. Yugal, 3. Gum, 4. Alveolar ridge, 5. Palate, 6. Retromolar, (iii) Clinical presentation (**pclinica**), with 4 levels: 1. Homogeneous, 2. Warty, 3. Erythro, 4. Lichen. (iv) Previous oral carcinoma (**carcin**), with 2 levels: 1. Yes, 2. No. (v) Smoker (a) (**tobacco**), with 4 levels: 0. NS/NC, 1. No, 2. Yes, 3. Ex-smoker. (vi) Smoker (**alcohol**), with 3 levels: 1. Yes, 2. No, 3. NS/NC. (vii) Clinical evolution in the studied period (**evo**), with 4 levels: 1. Stable, 2. Malignancy, 3. Recurrence. (viii) Number of lesions (**no**), which can be used as a nominal variable with 5 levels (each of them corresponds to the number of lesions the sample presents). There are other variables, such as Age (**age**), (iv) Lesion size (**size**), (v) Number of lesions, etc., that appear neither in Table 1 nor in Table 2 but that still played a clinical role in the statistical analysis.

### 2.6. Image Scanning and Analysis FD

For the digitization of the histological samples, we used a Leica SCN400F Microscope Slide Scanner (Leica Microsystems CMS GmbH, Wetzlar, Germany). The digital images were obtained with a Canon EOs 1300d camera, varying between 100 mm and 60 mm lenses from the same company, and a ring flash. For the processing and analysis of the images, we worked with two different software programs: qupath v0.3.2 (Quantitative Pathology and Bioimage Analysis, Center for Cancer Research and Cell Biology at Queen’s University, Belfast) and ImageJ with Zulu OpenJDK 13.0.6 (National Institutes of Health). For the process of applying the mask to select the different tissues, we used MATLAB R2018a.

In the histological images, we distinguished two well-differentiated study areas due to their completely different architectural compositions; on the one hand, epithelium, and on the other, connective tissue. Therefore, for each digitized slide, we obtained two values referring to each of the zones.

**Figure 1:**
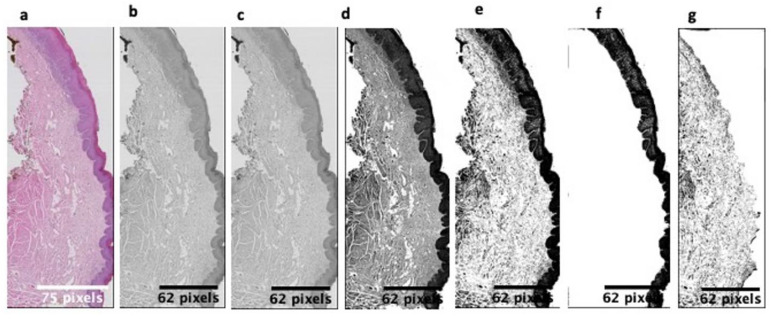
Image treatment process and level separation: (**a**) original image, (**b**) 8-bit image transformation, (**c**) threshold 128, (**d**) erosion and dilation, (**e**) make binary, (**f**) epithelium, cropped, and (**g**) connective tissue, cropped.

Using the first program, we selected the study area, extending it, once complete, to ImageJ, where we began to treat the images (Figure 1) following the White and Rudolph method for radiological images [17]. We automated the process with a macro performed ad hoc, to later mask the two differentiated sections (epithelium and connective tissue) and, thus, be able to calculate the value of ***dime*** and ***dimc***.

Once the masked image was obtained where we only had the epithelium or connective tissue, we began to apply the standardized method shown in Figure 1.

From the original image, we transformed it into an 8-bit image; after that, global thresholding at 128 was used (we used 128, as inspired by the White and Rudolph method; note that the FD values depended on the threshold value, but since it has been used for all images, and FD is an invariant of the structure, such an election does not affect the diagnostic value of FD). Later, we performed an erosion and dilation that allowed us to make the image binary. Finally, we cropped the epithelium and connective tissue images.

**Figure 2:**
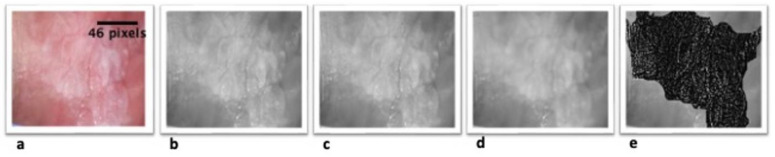
Binary process and filtering of the photographic image. (**a**) Digital cropped image of the buccal mucosa of a patient diagnosed with oral leukoplakia. (**b**) The cropped image, converted into an 8-bit image. (**c**) Image with added sharpening to make the lesion clearer, (**d**) Adding smoothing of the image. (**e**) A convolution filter was applied to define the limits of the lesion and crop the lesion image.

During the analysis of the digital photographic images obtained in the clinical examination, we also used both image programs as well as a segmentation process similar to that used for histopathological samples (Figure 2). Unlike in the previous process, we subjected the image to a series of filters that allowed us to delimit the lesion with absolute precision so that we could calculate the FD. On the initial image, we carried out two smoothing processes and applied a convolution filter, in order to delimit the lesion. With the limits of the lesion well defined, we created the mask that would help us to cut out the image, so that we were left with only the white lesion. Once this was complete, we applied the method previously described for the histological images.

To check the veracity of the calculated FD values, we again submitted the images, both micro- and macroscopic, for calculation of the value through the algorithm developed by the authors themselves, then we checked the values obtained with both methods. All measurements were performed by two examiners, applying Cohen’s kappa coefficient to the results obtained.

### 2.7. Statistical Analysis

The FD results were described as being in the median and interquartile range (IQR) or average and with standard deviation (SD). All statistical results were obtained in R (R version 3.6.3). Normality was verified using the Shapiro–Wilk test, then we applied the Wilcoxon/Mann–Whitney test, working at a 95% confidence level to make the comparison between quantitative variables (FD) and post hoc analysis, using Dunn’s test for variables with more than two categories. For comparisons involving both quantitative and qualitative variables, we used the Kruskal–Wallis and Wilcoxon tests for independent samples. For comparisons between the qualitative variables, the chi-square test and Fisher’s exact test were used. For the analysis of the FD of the photographic images and the relationship between age and FD, the correlation coefficient for non-parametric data was calculated.

For the study of the diagnostic performance, ROC curves were constructed to determine the area under the curve (AUC), the sensitivity, and the specificity. Differences were regarded as significant if the probability value *p* ≤ 0.05 and as highly significant if *p* ≤ 0.001.

## 3. Results

### 3.1. Description of the Sample

The sample consisted of a group of healthy subjects (*n* = 10) and another group of subjects with leukoplakia (*n* = 29). The average age was 61.2 ± 9.1 years for the NOM group and 68.9 ± 10.8 years for the OL group. After the statistics study, no significant differences appeared between age and the groups (*p* = 0.087), nor in terms of gender (*p* = 0.503). Table 1 shows all the clinicopathological variables of the study patients.

### 3.2. Analysis of FD

**Figure 3:**
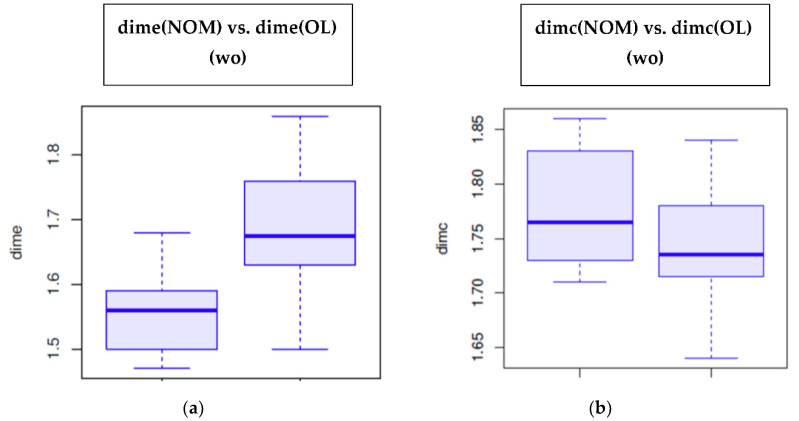
The values of: (**a**) ***dime*** OL vs. NOM; (**b**) ***dimc*** OL vs. NOM.

The values of ***dime*** and ***dimc*** are shown in Table 2. In the OL group, the median value of ***dime*** was higher (1.67, IQR = 0.12) than for the NOM group (1.56, IQR = 0.08), with statistically significant differences (Wilcoxon test, *p* = 0.0031) (Figure 3a). In relation to ***dimc***, the OL group presented values of 1.73, IQR = 0.06, while the NOM group presented values of 1.77, IQR = 0.06 (*p* > 0.05) (Figure 3b). There are no differences between ***dime*** and ***dimc*** in the OL group or the NOM.

### 3.3. Study of the Effect of the Types of Dysplasia on the Values of Dime and Dimc

We observed in the subgroup of patients without dysplasia that a mean ***dime*** value equal to 1.65 ± 0.10 was obtained. On the other hand, for those subjects with a low level of dysplasia, a mean ***dime*** of 1.64 ± 0.08 was obtained, while for the patients with a high level of dysplasia, a mean ***dime*** equal to 1.79 ± 0.08 was obtained (Table 3). Significant differences were observed between the non-dysplasia vs. high-grade (*p* = 0.0156) and low-grade vs. high-grade (*p* = 0.0049) groups. In relation to ***dimc***, there were no statistically significant differences for the different degrees of dysplasia. No differences were found between ages in the different subgroups of dysplasia, nor in the type of sample (*p* = 0.692 and *p* = 0.087, respectively). Moreover, no statistical correlation was found between the age and the *dime,* nor with ***dimc*** (*p* = 0.115 and *p* = 0.992, respectively). No differences were found between the genders and the ***dime*** or ***dimc*** (*p* = 0.797 and *p* = 0.223, respectively).

### 3.4. Diagnostic Yield Analysis

The best cut-off point for the diagnostic value of the ***dime*** FD was determined. For a cut-off point of 1.44 of ***dime***, a sensitivity of 96.6% was obtained, with a specificity of 100% and an AUC = 0.819 (*p* = 0.003) (Figure 2).

### 3.5. Pilot Study of FD in Clinical Images and Its Relationship with Dysplasia

As a pilot test, the FD analysis was performed on the photographic images of OL lesions (*N* = 17) prior to performing a diagnostic biopsy using two different protocols (macro 1 and macro 2). For macro 1, an FD of 1.81 ± 0.81 was obtained, while for macro 2 an FD of 1.80 ± 0.76 was obtained. Between these two methods, a Spearman correlation coefficient of 0.879 (*p* < 0.0001) was obtained. For macro 1, it was established that the lesions reduced their DF as the degree of dysplasia increased; the values of OL without dysplasia (FD = 1.85 ± 0.09), low-grade dysplasia (1.82 ± 0.04), and high-grade dysplasia (1.71 ± 0.75) showed significant differences between the groups without dysplasia and high-grade dysplasia (Bonferroni, *p* = 0.045).

## 4. Discussion

The criteria used for the diagnosis of epithelial dysplasia in the oral cavity include, as in other mucosae, both cytological and architectural alterations. The degree of dysplasia continues to be one of the most reliable markers today for determining the risk of the malignant transformation of OPMD [22]. The 2005 WHO classification recognized 5 histopathological categories by which to classify precursor epithelial lesions: squamous cell hyperplasia, mild dysplasia, moderate dysplasia, severe dysplasia, and carcinoma in situ [23]. In the 2017 classification, the WHO recognizes that dysplasia grading is poorly reproducible between observers, and simplifies its classification by eliminating carcinoma in situ, considering it synonymous with severe dysplasia [24]. In the same line of criteria simplification, in 2008, Warnakulasuriya et al. proposed a binary system with 2 categories of low-grade and high-grade dysplasia, eliminating those classifications of moderate degree in order to facilitate the categorization of the lesions [25]. However, Kujan was the first author to introduce the concept of a binary system regarding architectural and cytological changes [8].

This presence of the different classification systems, together with the inherent subjectivity of some histological parameters, caused great inter-examiner variability and a lack of standardization in the diagnosis of epithelial dysplasia [26], which, in our opinion, should be corrected. In this sense, the search for altered morphological patterns that can be detected by mechanized and standardized reproducible methods, other than the human eye, could be of great help in diagnosis [27,28,29,30]. Many studies have been carried out, trying to find quantifiable signs that could help us to evaluate the malignant capacity of OLs. FD has been the subject of many of these studies, applying such values to the study of pathological anatomy samples, as well as to photographs of the clinical lesion itself [31,32,33].

Landini et al. carried out various studies where they used the FD to analyze several samples of leukoplakia in its different degrees, as well as healthy tissues and tissues with oral squamous cell carcinoma, giving special importance to what they called the epithelium–connective tissue interface (ECTI). In their work, results were obtained with significant differences between the different groups that made up the sample in the specific study areas (ROI), obtaining what they called local FD values [34].

The novelty of our study is that we were able to calculate the FD value in all the tissues that made up the samples analyzed and stained with hematoxylin-eosin. We segmented two areas that were clearly distinguishable at the compositional level and calculated the FD value of each one: both epithelium and connective tissue. The interpretation of the results obtained showed a difference in FD values between those samples obtained for epithelium and connective tissue. Previous studies conducted at the radiological level, where the normal ranges for this invariant were stated, proved that this technique can also be extrapolated to pathological anatomy samples [35].

The increase in FD obtained in cases of severe dysplasia is translated into the loss of the architecture of the epithelium when the sample becomes more disorganized. If the value of the FD decreased below the established range, we could not consider this type of sample to be a real fractal, since the definition of fractal structure and the ranges established by the authors at a radiological level indicate that very low levels do not recognize strata and are close to what would be the equivalent of white (a line) at the mathematical level.

The analysis of the FD regarding the clinical photographs taken during the examination by the dentist in charge of the diagnosis and monitoring of the lesions is considered. In this sense, this study is a seminal work, providing a deeper approach than those performed in previous works that were conducted without distinguishing the layers of completely different architecture.

Iqbal et al. [36] determined the efficacy of FD analysis in detecting the malignant potential of OL in digital imaging and the results were compared via biopsy. The FD values were significantly higher in OL with dysplastic changes, as happened in our study. In this work, it was reported that the FD values increased as the age of the patients increased. Along the same lines, Jurczyszyne et al. [37] reported that in OL patients treated with a LiteTouch™ Er: YAG dental laser, the FD in the photographic images increased significantly after laser treatment, allowing FD to be used as a control mechanism for the effectiveness of the treatment.

The fractal dimension is a mathematical value that is common for each structure and measures in some way the internal similarity of such an object, particularly in the case of the biological structures used in the study for the present work. The fractal dimension in the current work has been computed via the so-called “box-counting technique”, modified using our algorithm, which was introduced in [38].

Healthy human tissue has a dense internal similarity at the micro-level; when a pathological process occurs, this internal coherence is broken down, with holes or a bigger cell structure appearing. The fractal dimension of a plane image such as the ones we have analyzed will be between 1 and 2. Note that the healthy areas reach values from 1.6 or 1.7 and all separation values from such a level will mean non-normality, the worst values being those closest to the interval (1, 2) boundaries.

In our study, we have found that the normal oral mucosa presents a lower FD than oral leukoplakia. Regarding the degree of dysplasia, in low-grade dysplasias, we found that they presented a DF value similar to that of the normal oral mucosa; this can be explained by the fact that low-grade dysplasias are frequently associated with regenerative changes to the epithelium, which implies a differentiation of the epithelium toward a more normal structure. However, high-grade dysplasias are related more closely to the processes of de-differentiation toward malignant processes; for this reason, the DF values increase significantly in high-grade dysplasias compared to low-grade ones.

The limitations of this study were derived mainly from the small sample size and the lack of clinical image standardization, which is primarily linked to the position, angle, and focus of the image on the different locations in the oral cavity.

## 5. Conclusions

It can be concluded that the FD of OL lesions is different in relation to NOM samples, especially at the level of the epithelium, achieving high diagnostic sensitivity as a diagnostic test. These differences in ***dime*** are more pronounced in those lesions with a high degree of dysplasia. The FD of a healthy area reaches values from 1.6 or 1.7; all deviations in value from such a level will mean non-normality; normal oral mucosa presents a lower FD than oral leukoplakia. In relation to the degree of dysplasia, low-grade dysplasia yields values similar to that of normal oral mucosa.

Regarding the study of FD in photographic images, both methods of obtaining values seem to correlate well, allowing the researcher to establish differences in FD in the different levels of dysplasia.

## Figures and Tables

**Table 1:** Clinical and pathological characteristics of OL and NOM samples.

OL Group		*N*	Ratio	Gender Distribution	
				Men *N* (Ratio)	Women *N* (Ratio)
Gender	Men	11	38		
	Women	18	62		
	Total	29	100		
Level of dysplasia	No dysplasia	13	45	6 (55)	7 (39)
	Low	11	38	3 (27)	8 (44)
	High	5	17	2 (18)	3 (17)
	Total	29	100	11 (100)	18 (100)
Biopsy location	Tongue	14	48	6 (55)	8 (44)
	Buccal mucosa	8	28	3 (27)	5 (28)
	Gum	1	3	0 (0)	1 (6)
	Alveolar ridge	2	7	1 (9)	1 (6)
	Palate	2	7	1 (9)	1 (6)
	Retromolar	2	7	0 (0)	2 (11)
	Total	29	100	11 (100)	18 (100)
Clinical presentation	Homogeneous	15	52	8 (73)	7 (39)
	Warty	9	31	2 (18)	7 (39)
	erythrocyte	3	10	1 (9)	2 (11)
	Lichenoid	2	7	0 (0)	2 (11)
	Total	29	100	11 (100)	18 (100)
Previous oral carcinoma	Yes	5	17	5 (45)	0 (0)
	No	24	83	6 (55)	18 (100)
	Total	29	100	11 (100)	18 (100)
Clinical evolution	NR/DK	2	7	0 (0)	2 (11)
	Stable	18	62	8 (73)	10 (56)
	Malignancy	8	28	2 (18)	6 (33)
	Recurrence	1	3	1 (9)	0 (0)
	Total	29	100	11 (100)	18 (100)
Smoker	NR/DK	5	17	1 (9)	4 (22)
	No	15	52	2 (18,2)	13 (72)
	Yes	3	10	3 (27,3)	0 (0)
	Ex-smoker	6	21	5 (45,5)	1 (6)
	Total	29	100	11 (100)	18 (100)
Alcohol consumer	NR/DK	9	31	4 (36,5)	5 (28)
	No	16	55	4 (26,5)	12 (66)
	Yes	4	14	3 (27)	1 (6)
	Total	29	100	11 (100)	18 (100)
**NOM Group**		***N***	**Ratio**	**Gender Distribution**	
				**Men** ***N*** **(Ratio)**	**Women** ***N*** **(Ratio)**
Gender	Men	5	50		
	women	5	50		
	Total	10	100		
Receives medication of some kind	Yes	2	20	1 (20)	1 (20)
	No	8	80	4 (80)	4 (80)
	Total	10	100	5 (100)	5 (100)
Smoking	Yes	4	40	2 (40)	2 (40)
	No	6	60	3 (60)	3 (60)
	Total	10	100	5 (100)	5 (100)

**Table 2:** Fractal dimension for ***dime*** and ***dimc*** in the OL and NOM groups.

	NOM (*N* = 10)				OL (*N* = 29)			
	Mean	Std	Median	IQR	Mean	Std	Median	IQR
dime	1.56	0.07	1.56	0.08	1.67	0.10	1.67	0.12
dimc	1.78	0.06	1.76	0.09	1.74	0.06	1.73	0.06

**Table 3:** Fractal dimension for dime and dimc according to the degree of dysplasia in the OL group.

	Level 1 without Dysplasia (Count = 13)				Level 2: Low Level of Dysplasia (Count = 11)				Level 3: High Level of Dysplasia (Count = 5)			
	Mean	Std	Median	IQR	Mean	Std	Median	IQR	Mean	Std	Median	IQR
***dime***	1.65	0.1	1.68	0.07	1.64	0.08	1.65	0.12	1.79	0.08	1.82	0.05
***dimc***	1.75	0.07	1.76	0.08	1.73	0.06	1.72	0.05	1.74	0.04	1.73	0.05

## Data Availability

The data used in this study are contained in this manuscript.
